# Income of informal labourers in rural areas: A survey dataset of northern mountainous regions in Vietnam

**DOI:** 10.1016/j.dib.2021.107292

**Published:** 2021-08-14

**Authors:** Anh Ngoc Mai, Thao Huong Pham, Ha Thanh Hoang, Hoa Hoang Thi Nguyen

**Affiliations:** a Faculty of Management Science, National Economics University, Hanoi, Vietnam; b Research Management Department, National Economics University, Hanoi, Vietnam; c Academic Affairs Office, National Economics University, Hanoi, Vietnam; d Faculty of Real Estate and Resources Economics, National Economics University, Hanoi, Vietnam

**Keywords:** Income, Education, Vocational training, Credit accessibility, Technological application, Rural labourers

## Abstract

This article introduces a dataset that presents not only the income of informal labourers in Vietnam's northern mountainous regions but also rural labourers' characteristics and the factors affecting rural labourers' income. The data collection was conducted in five provinces: Tuyen Quang, Quang Ninh, Ha Giang, Yen Bai, and Bac Giang. Farmers and labourers without labour contracts in rural regions are the target groups. A total of 750 survey questionnaires were equally distributed across five provinces through the snowball sampling method; in all, data from 725 collected questionnaires were selected for analysis. The dataset was collected through a three-part survey. Nominal, ordinal, and scale were used in the survey questionnaire. Tables and bar charts present data outputs. Analysis of the data provided insight into the correlation between the rural labourers' income and factors that may prove beneficial for researchers and provincial policymakers interested in, and in charge of, social development in rural areas. The dataset was obtained as a reference source for intensive studies on socio-economic development in rural regions.

## Specifications Table

**Table:** 

Subject	Economic Development and Growth
Specific subject area	Factors affecting income of informal labourers in rural areas
Type of data	Table Bar Chart Text document
How data were acquired	Questionnaire survey
Data format	Raw and analysed data Descriptive and statistical data
Parameters for data collection	Participants are informal labourers living in five provinces of Vietnam's northern mountainous regions; all voluntarily participated in the survey; Nominal, ordinal, and scale.
Description of data collection	Data were gathered through questionnaires distributed to informal labourers living in Vietnam's northern mountainous regions using snowball sampling. The surveys were conducted through face-to-face interviews with the research team, assisted by several local officials from five provinces. Data were screened for missing values and eliminating bias answers. Outliers were checked before pursuing data analysis. The final sample size consists of 725 valid responses.
Data source location	Five provinces in Vietnam's northern mountainous regions, including Tuyen Quang, Quang Ninh, Ha Giang, Yen Bai, and Bac Giang.
Data accessibility	Data are included in this paper and in Mendeley Data: http://dx.doi.org/10.17632/s6p7dwj3j6.1

## Value of the Data

• The dataset provides essential information for exploring the correlation between the informal rural labourers' income and several factors.
• The dataset is crucial not only for researchers but also for policymakers and local officials who are dealing with socio-economic development and trying to find ways to improve the living conditions of informal rural labourers in Vietnam's northern mountainous regions. The dataset also presents essential insights for non-governmental organisations to optimise influenced factors towards poverty alleviation programmes.
• The dataset will encourage intensive research on rural labourers’ quality of life or labourers’ assessments of conditions for socio-economic development of five rural sample provinces, etc.

## 1 Data Description

The survey data file spreadsheet accompanying this article consists of 725 rows and 29 columns. Each row presents an individual's response to the questionnaire. Each questionnaire item in the columns was assigned a label as shown in the first row: *C* is the short form for characteristics; *T* for all kinds of incomes; *F* for impact factors; *L* for living conditions; *P* for respondents’ opinions on policies; and A for respondents’ assessment on current issues in rural areas (see Table 1). Items are measured by nominal, ordinal, or scale. While variables belong to *C, T* and *F* will be analysed in depth for this study. *L, P*, and *A* variables will be presented in incoming intensive research.

**Table 1:** Label of data.

Constructs	Items	Code	Measure
	Provinces	CPRO	Nominal
	Gender	CGEN	Ordinal
Characteristics	Race	CRAC	Ordinal
	Careers	CJOB	Nominal
	Income quintile group	CQUI	Nominal
	Total income	TEIN	Scale
Income	Agricultural income	TAIN	Scale
	Service and industrial income	TSII	Scale
	Others	TOIN	Scale
	Education	FEDU	Nominal
Factors	Participating in vocational training courses	FVTP	Ordinal
	Credit's accessibility	FCRA	Ordinal
	Technological application	FTAP	Ordinal
	Cottage house	LHO1	Ordinal
Living conditions	Roofed house	LHO2	Ordinal
	Solid house	LHO3	Ordinal
	Buildings	LHO4	Ordinal
	Real loan	LCRE	Scale
	Saving	LSAV	Scale
	Working days	LWDA	Scale
	Training Qualification	PPO1	Nominal
	Supporting Chains for agricultural activities	PPO2	Nominal
Opinions on Policies	Healthcare	PPO3	Nominal
	Taking care toward Elderly	PPO4	Nominal
	Obtaining Urban jobs	PPO5	Nominal
	Increased woman labour in rural areas	ARO1	Nominal
	Increased Woman roles in their families	ARO2	Nominal
Personal Assessment	Elderly responsibility	ARO3	Nominal
	Better living conditions in rural areas	ARO4	Nominal
	The higher technique of returnee labourers	ARO5	Nominal

*Source:* Authors' survey.

Table 2 shows 725 respondents' characteristics in five selected provinces in the northern mountainous region of Vietnam. This region is known as a nationally important geographical and political feature because of various ethnic minorities living together and because it adjoins China and Laos. The dataset has 725 observations, of which 81.7 and 46.3% are male and King people, respectively. Although rural labourers carry out agricultural or/and non-agricultural economic activities, the most significant portion of the respondents' careers relates to agricultural activities, accounting for 71%, and the lowest proportion of the respondents' careers is working in the service and industrial sectors. Regarding the quintile, 4.1 and 14.1% of the respondents belong to the top quintile, the fourth quintile. Most respondents belong to the middle quintile and 16.3 and 22.6% relate to the second and lowest quintiles, respectively.

**Table 2:** Respondents' characteristics.

		Frequency	Percent	Valid Percent	Cumulative Percent
Gender	Male	592	81.7	81.7	81.7
	Female	133	18.3	18.3	100.0
	Total	725	100.0	100.0	
Race	King	336	46.3	46.3	46.3
	Minorities	389	53.7	53.7	100.0
	Total	725	100.0	100.0	
Careers	Working in the agricultural sector	515	71.0	76.2	76.2
	Working in the service and industrial sector	62	8.6	9.2	85.4
	Others	99	13.7	14.6	100.0
	Total	676	93.2	100.0	
Missing	System	49	6.8		
Total		725	100.0		
Income quintile group	Top quintile	30	4.1	4.2	4.2
	Fourth quintile	102	14.1	14.1	18.3
	Middle quintile	307	42.3	42.6	60.9
	Second quintile	118	16.3	16.4	77.3
	Lowest quintile	164	22.6	22.7	100.0
	Total	721	99.4	100.0	
Missing	System	4	.6		
Total		725	100.0		

*Source:* Authors' survey.

Table 3 illustrates the total income of surveyed rural labourers. In the educational qualification aspect, higher educational qualifications do not guarantee rural labourers that their minimum internal income will be consistently higher than that of a rural labourers' educational qualifications. However, the descriptive statistics show that the maximum internal revenue of rural labourers with higher academic qualifications is better than those of those possessing lower educational qualifications.

**Table 3:** Descriptive Statistics about the total income of sample rural labourers.

					Mean		

		*N* Statistic	Minimum Statistic	Maximum Statistic	Statistic	Std. Error	Std. Deviation Statistic
Primary education	Total income	473	7	86	29.37	.61	13.31
Lower secondary education	Total income	136	17	150	42.94	1.84	21.42
Upper secondary education	Total income	73	13	180	60.08	3.54	30.21
Others	Total income	19	20	560	132.08	27.54	120.02
Short courses	Total income	609	7	150	324	102	1648
Long Courses	Total income	92	13	560	7495	230	6656
Credit Inaccessibility	Total income	635	0	560	3794	131	3291
Credit accessibility	Total income	87	8	124	3836	2,60	2424
Low technique	Total income	296	3	150	3266	102	1750
Adequated techniques	Total income	327	8	560	4201	230	4166

*Source:* Authors’ survey.

Without the upper secondary education certificate, most rural labourers take part in short vocational training programmes; their incomes are relatively low compared to those participating in the longer courses. The table also indicates that the differences in technological application in the daily working of surveyed labourers are minor. Although the gap of rural labourers' maximum income between the two groups is broad, the gap of rural labourers' average income is not high.

## 2 Experimental Design, Materials and Methods

### 2.1 Questionnaire design

The survey questionnaire was developed from previous studies, and it has been adapted for use in the context of Vietnam. The questionnaire consists of three parts.

The first part refers to questions on the characteristic profile of the interviewees. Respondent's province, gender, race, career, and quintiles were included in the questionnaire. The questionnaire uses nominal and ordinal data for collecting information.

The second part consists of questions on respondents' income and several factors influencing internal incomes.

The income of informal labourers in rural areas is generated from remuneration for labourers' participating in agricultural and non-agricultural activities [6]. Without having labour contracts, the income becomes the primary source of informal labourers' living [4]. Scale data were used to collect information related to incomes from participating in agricultural activities (Mil VND), incomes from participating in service or/and industrial activities (Mil VND), and other incomes (Mil VND), as well as total incomes (Mil VND).

The income of informal labourers is affected by factors that include educational qualification, vocational training, credit accessibilities, and technological application.

Education refers to the school years of educated people. The higher educational qualification that a labourer acquires, the better knowledge a labourer possesses. Knowledge is a prerequisite for improving working skills, thereby resulting in higher productivity and better income [1,2]. Nominal data were used to collect information. Respondents ticked one of the educational qualifications boxes, choosing from primary education, lower secondary education, upper secondary education, or others.

Both short and long courses of vocational training programmes improve learners' knowledge and skills. The more skills a labourer archives, the higher income a labourer obtains. While the short courses focus on livelihood programmes based on the design of on-the-job training or learning, the long courses, held at vocational training schools, enhance the chances for graduates to seek non-agricultural employment [2]. Ordinal data were used to collect information. Respondents indicated the types of vocational training courses in which he/she had participated.

The rural labourers' savings are relatively limited [7]. They do not have money to re-invest in farming and non-farming activities. Therefore, low and inadequate incomes are inevitable. Unless obtaining loans from Bank for Social Policies for upgrading technologies, their productivity would be improved, and their income would be increased [3,4]. Ordinal data were used to collect information. Respondents informed whether he/she accessed credit from the Vietnam Bank for Social Policy.

Thanks to technological application to the production process, jobs have been conducted with higher productivity. Labourers can engage in other employment to earn additional income [2]. Ordinal data were used to collect information. Respondents ticked the boxes for low or adequate technique.

The final part includes questions on personal opinions about supporting policies, living conditions, employment opportunities, etc. Ordinal data were used. Respondents shared the state of their agreement or disagreement through a 5-point Likert scale, where 1 = strongly disagree; 2 = disagree; 3 = neutral; 4 = agree; and 5 = strongly agree).

The researchers consulted experts from central and local governments for questionnaires and data output.

### 2.2 Data collection

The snowball sampling method was chosen. Snowball sampling or chain-referral sampling is defined as a non-probability sampling technique. This method is particularly useful for finding people who are unwilling to reveal their identities. Snowball sampling provides the researcher the opportunity to communicate better with the samples [5]. The researcher gains a deeper and more thoughtful understanding of the informal workers' incomes in Vietnam's northern mountainous areas and this target group's perception of the factors affecting their incomes.

Under the support of the Department of Labour, War invalid, and Social affairs in five selected provinces, target groups were identified for the snowball sampling survey. Most of the chosen groups are poor and living in mountainous areas. The heads of the target groups acted as preliminary samples because they can introduce potential respondents who fit the requirements of the survey based on their understanding of local labourers' employment and income. New samples were recruited from the acquaintances of preliminary samples. This process will continue in a semi-automatic and chain-like manner until data saturation of around 150 questionnaires per province is reached; however, it differentiates from province to province due to differentiation in geographical allocation, the participants’ willingness, and the interviewers' expectations.

A total of 750 survey questionnaires were distributed equally to interviewees from five provinces. The survey was conducted directly at the informal rural labourers' residences or at their place of work. An informal rural labourer received the payment of 3 USD after completing the questionnaire, and participants were informed that the survey was anonymous. Seven hundred twenty-five survey questionnaires were collected, then the data were re-categorised and recoded for questions concerning the income of informal rural labourers. The final step in processing was to output the data, such as descriptive tables, frequency tables, and bar charts. Cross-tabulation was also used to explore the correlation between informal rural labourers' income and several chosen factors.

## Ethics Statement

Informed consent was obtained from all participants before participation, and a full debriefing followed after the study was completed. Participants were informed that the survey was anonymous.

## CRediT Author Statement

**Anh Ngoc Mai:** Conceptualization, Methodology, Software, Writing – reviewing & Editing; **Thao Huong Pham**: Methodology, Writing – Original draft, Data curation, Writing – review & editing; **Ha Thanh Hoang**: Writing – original draft, Writing – reviewing & editing; **Hoa Hoang Thi Nguyen:** Writing – original draft, Writing – reviewing & Editing.

## Declaration of Competing Interest

The authors declare that they have no known competing financial interests or personal relationships which have or could be perceived to have influenced the work reported in this article.
